# Preoperative Embolization of Fisch Grades II–IVa Juvenile Nasopharyngeal Angiofibromas: Transarterial Embolization in the Age of Onyx

**DOI:** 10.7759/cureus.15804

**Published:** 2021-06-21

**Authors:** Kunal Vakharia, Jaims Lim, Muhammad Waqas, Michael K Tso, Elad I Levy, Adnan H Siddiqui, Jason Davies

**Affiliations:** 1 Department of Neurosurgery, Mayo Clinic, Rochester, USA; 2 Neurosurgery, Jacobs School of Medicine and Biomedical Sciences, University at Buffalo, Buffalo, USA

**Keywords:** juvenile nasopharyngeal angiofibroma, onyx, transarterial embolization, tumor devascularization

## Abstract

Objective

The current mainstay treatment for juvenile nasopharyngeal angiofibromas (JNAs) is surgical resection, but embolization of primary feeding arteries through endovascular transarterial and direct tumoral puncture embolizations with various agents has been described. We describe a single institutional experience with JNA embolization utilizing Onyx (Medtronic, Dublin, Ireland).

Methods

A retrospective records review was performed to identify patients who underwent embolization for devascularization of Fisch grades II-IVa JNA (tumor extension beyond the sphenopalatine region) before surgical resection between 2010 and 2019. Fluoroscopy time, grade, intraoperative blood loss, and clinical follow-up data were compiled. Tumor devascularization percentage was calculated using ImageJ software (public domain, BSD-2) by measuring the ratio of preoperative and postoperative embolization tracing.

Results

Five consecutive patients (ages 12-16 years [average 14 years]; all male) with JNAs underwent preoperative transarterial embolizations performed under general anesthesia. All patients presented with epistaxis; two also presented with headaches. Fisch grades were II in two patients, IIIa in two, and IVa in one. The patient with the grade IVa lesion underwent direct transtumoral puncture and Onyx embolization. The mean percentage of all tumor devascularizations postembolization was 86.0±9.7%.Complete resection 24-48 hours postembolization was obtained for grades II and IIIa lesions with <700 mL blood loss. No embolization-related complications and no clinical sequelae were present in the five cases after embolization.

Conclusion

In our experience, Onyx embolization of JNAs was safely conducted with adequate tumor penetration beyond the sphenopalatine region through transarterial routes.

## Introduction

Juvenile nasopharyngeal angiofibromas (JNAs) are relatively rare, accounting for 0.05% of all head and neck tumors [1]. These tumors typically affect adolescent males and present with nasal obstruction and recurrent and profuse epistaxis [2]. They typically arise from the lateral wall of the nasopharynx at the superior margin of the sphenopalatine foramen and are typically categorized based on the Fisch classification, depending on the extent of involvement beyond the sphenopalatine foramen [3]. The Fisch classification is a classification system based on the pattern of tumor spread that was proposed by Andrews et al. [4]. Based on the region and extent of tumor infiltration, a grade of I-IVb is assigned, with the higher grades indicating more extensive tumor invasion. Although many different approaches to the management of these tumors have been described, endoscopic surgical resection is typically the most widely accepted approach for lesions that do not have infratemporal fossa or intracranial extension. Because of the high vascularity of these tumors, embolization has been utilized to help control intraoperative blood loss and aid in reducing operative time, resection planes, and hemostasis [2].

Because of the complex neovascular architecture, along with the possible extracranial-to-intracranial anastomosis that can be present in patients with JNA, several therapies have focused on preoperative embolization, including both transarterial and direct tumoral puncture approaches [5]. There have been several descriptions of transarterial embolization procedures for these tumors using Gelfoam (Pharmacia and Upjohn, Kalamazoo, MI) [6], polyvinyl alcohol (PVA) [7], coils [8], n-butyl cyanoacrylate (NBCA) [9], and Onyx (Medtronic, Dublin, Ireland) [10]. Particularly with the use of smaller particles, anastomoses that are not readily apparent can inadvertently be embolized, leading to complications including central retinal artery occlusions, strokes, and embolization of parent external carotid artery (ECA) branches [11].

Although the mainstay treatment for these lesions is surgical resection, understanding the goals of care and the safest means to achieve adequate resection can be challenging. Many otolaryngologists resect tumors that are primarily restricted to the nasopharynx or with limited extension beyond the sphenopalatine region. With extension into the maxillary region, infratemporal fossa, and with an intracranial extension of both the tumor and associated vascular feeders, these lesions pose a considerable challenge [12-15]. In one series, the results for five patients treated with transarterial embolization with Onyx were compared to those for five patients treated with direct tumoral embolization [16]. The direct tumoral puncture was associated with shorter embolization times and a higher degree (93% vs. 77%) of devascularization [16]. For many patients undergoing either transarterial or direct tumor embolization, the primary focus is on embolization of the nidus of the tumor contained within the sphenopalatine region. The Fisch classification helps delineate JNA contained in the nasopharynx, those that extend to the pterygomaxillary fossa, those with infratemporal extension, and those with intracranial extension and vascular supply [13]. Many of the current descriptions of direct tumoral embolization focus on lesions that are primarily visible upon endoscopic inspection for direct puncture including Fisch grades I and II lesions [16,17]. We present our series of Fisch grades II-IVa presurgical embolizations with Onyx, an embolic agent with good controllability and redirectability, along with postsurgical outcomes.

## Materials and methods

Study population and data collection

After receiving institutional review board approval, we retrospectively reviewed the medical records for all patients with Fisch grades II-IVa JNAs embolized with Onyx at our institution over the past nine years (between 2010 and 2019). Patients with JNAs undergoing embolization with Gelfoam or PVA were excluded. Data that were collected included fluoroscopy time, percentage of tumor devascularization, Fisch classification, perioperative complications, intraoperative blood loss, and follow-up (length of follow up and clinical status). The percentage of tumor devascularization was calculated using ImageJ software (public domain, BSD-2) [18] and measuring the ratio of pre- to postembolization tracing on this software. All embolizations were carried out prior to surgical resection.

All patients who underwent embolization were presented at a multidisciplinary vascular conference as part of routine preparation for complex vascular cases. All decisions to embolize lesions were made after discussion of all possible surgical options, and decisions regarding transarterial versus direct tumoral embolization were chosen based on embolization of difficult-to-access portions of the tumor. All surgeons involved in embolization procedures were dual-trained cerebrovascular surgeons.

Transarterial embolization technique

Informed consent was obtained from the patients’ healthcare proxies before procedures were performed. Procedures were performed under general anesthesia. Patients received heparin pre-procedurally until an activated clotting time between 250 and 300 seconds was achieved after arterial access was obtained. A 6-French Envoy guide catheter (Codman Neuro-Integra LifeSciences, Raynham, MA) was used for both common carotid artery injections and then navigated into the origin of the ECA. Feeding vessels into the nidus of the tumor were selectively catheterized using a Headway Duo 167cm microcatheter (MicroVention-Terumo, Aliso Viejo, CA) and a Synchro2 microwire (Stryker Neurovascular, Fremont, CA). Microcatheter angiography was performed to demonstrate that the catheter was within the body of the nidus in order to allow for sufficient penetration of the Onyx 18 and limit inadvertent embolization to collateral vessels. Dimethyl sulfoxide (DMSO) was used to coat the inner diameter of the microcatheter and was slowly injected until the entire volume of the microcatheter was filled. Negative roadmap guidance was used to ensure that the embolic material was within the confines of the tumor vascular blush with allowable reflux into the supplying parent pedicle. When there was a concern for an extension to dangerous areas of anastomoses near-normal vasculature, the injection was halted for a period of 1 minute and then restarted. This allowed serial assessment of embolization progress and identification of key collateral vasculature through guide catheter angiographic runs. Tumor penetration was achieved best with a proximal pedicle plug that allowed the Onyx to travel into new nidal vessels more easily and prevented reflux of the Onyx backwards, concomitantly limiting forward flow and occlusion of targeted vessels. Suction with a 1 mL syringe was used to aspirate the microcatheter as it was removed. Final transarterial postembolization runs were performed. Of note, multiple microcatheters were used in more complex lesions to allow for different pedicles to be sufficiently embolized and to limit the amount of radiation at one location.

For direct tumoral embolization, an 18-gauge metal spinal needle was used for a direct transnasal puncture. A 20-cm length of extension tubing was connected to the needle and direct insertion into the JNA was confirmed with contrast injection. DMSO and Onyx 18 were injected until approximately 50% of the vascular territory initially visualized with contrast injection within the tumor was devascularized.

## Results

A total of five patients with JNAs underwent preoperative transarterial embolization (ages 12-16 years [average 14 years]; all male) (Table 1).

**Table 1:** Patient background and demographics

Patient	Age (years)	Sex	Fisch Grade	Presenting Symptom	% Devascularization	Complications	Time from Embolization to Surgical Resection (days)
1	12	M	2	Epistaxis	71	None	1
2	16	M	3a	Epistaxis, lightheadedness	93	None	1
3	15	M	3a	Epistaxis, headaches	96	None	2
4	14	M	2	Epistaxis	84	None	1
5	13	M	4a	Epistaxis, headaches, & oral bleeding	86	None	Staged

All patients presented with epistaxis; two presented with headaches and one with light-headedness. The Fisch grades were II in two patients, IIIa in two patients, and IVa in one patient. A total of 11 vessels were embolized in the five patients. Onyx 18 was used in all cases. All Fisch grade II and IIIa lesions were resected 24-48 hours after Onyx embolization with an estimated blood loss of 700 mL in each case. Mean tumor devascularization for all lesions was 86±9.7%. The extent of devascularization for each lesion is provided in the table. Mean fluoroscopy time for Fisch grades II and IIIa lesions was 54±8.2 minutes. After preoperative embolization, residual vascularization of the tumor was present in all five cases, particularly in the intracranially extending portions of the JNA. There were no neurological complications related to any of the embolization procedures. Inadvertent embolization of parent vessels occurred in the ECA in one grade IIIa and the one grade IVa lesion with no related complications. Complete resection was obtained in the grade II and grade IIIa lesions.

**Figure 1:**
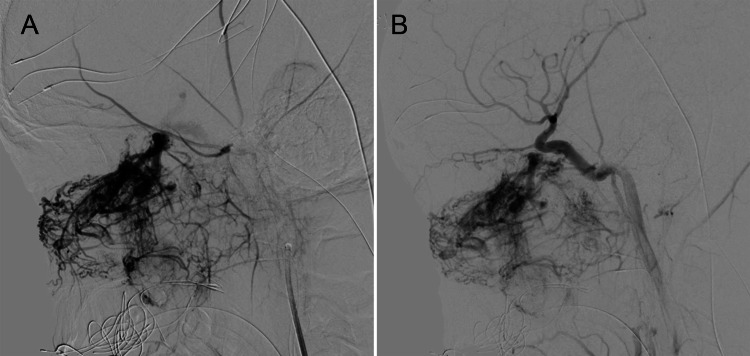
Transarterial embolization of Fisch grade IVa juvenile nasopharyngeal angiofibroma Serial lateral left external carotid artery injections demonstrate pre-embolization (A) and post-embolization (B) transarterial filling of a Fisch grade IVa juvenile nasopharyngeal angiofibroma of the left nasal cavity. Decreased vascularization is seen secondary to embolization.

**Figure 2:**
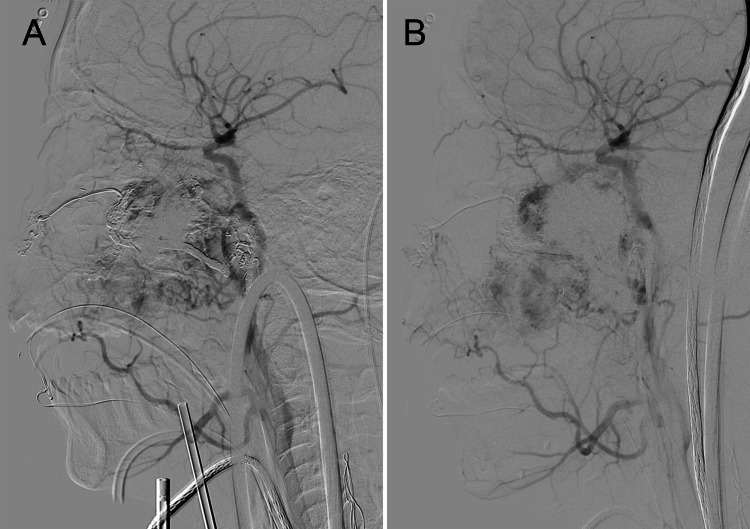
Direct transnasal puncture embolization of Fisch grade IVa juvenile nasopharyngeal angiofibroma of left nasal cavity Serial lateral left common carotid artery injections demonstrate pre-embolization (A) and post-embolization (B) arterial filling of a Fisch grade IVa juvenile nasopharyngeal angiofibroma of the left nasal cavity during direct transnasal puncture embolization. Decreased vascularization is seen secondary to embolization.

The single grade IVa lesion included in this study required a staged approach with three sessions, each separated by three months. The first two stages focused on an endoscopic transnasal approach for tumor debulking, and the third focused on the resection of intracranially extending portions of the mass through a dual endoscopic and open approach via a medial maxillectomy. The patient underwent two preoperative transarterial embolizations for the first two surgical resections. During the second surgical resection, direct tumoral puncture embolization was also performed intraoperatively under fluoroscopic guidance prior to actual surgical resection. The pre- and postembolization images of both the transarterial and direct tumoral puncture approach are seen in Figures 1A, 1B and 2A, 2B. Complete resection was achieved for the intracranial portion and sphenopalatine region of the tumor, but a portion of the infratemporal extension was left in place.

There were no complications after embolization or surgical resection in the five patients. Follow up for the four patients with Fisch grades II and IIIa lesions after surgical resection ranged from 13 days to 1,689 days. All four patients were doing well at their last follow up without complications or neurological deficits. The patient with the Fisch grade IVa lesion is being serially monitored and was found to have recurrent growth of the lesion, with surgery planned in the future.

## Discussion

An understanding of the angioarchitecture surrounding complex vascular tumors of the head and neck is critical for surgical planning as well as preoperative embolization. JNAs are relatively rare lesions and often affect male adolescents [2]. These patients typically present with nasal obstruction and epistaxis. The tumor originates from the lateral wall of the nasopharynx but can extend into the pterygomaxillary region, infratemporal region, and have intracranial extension. To categorize the staged approach for surgical management of these lesions, the Fisch classification subdivides tumors that extend across multiple regions [3].

Types of embolizations

Because JNAs are highly vascular lesions, those lesions that are contained within the nasopharynx and sphenopalatine region can be embolized through a direct tumoral puncture or a transarterial route. Elhammady et al. demonstrated that embolization of these tumors can be done efficiently with a direct puncture with a decreased fluoroscopy time, better tumor devascularization, and decreased estimated blood loss [16]. Rosenbaum-Halevi et al. demonstrated efficient embolization of JNAs through dual access of both the ECA and the internal carotid artery (ICA) [19]. A 6F catheter was placed in the ECA with subsequent injection of Onyx 34 into the tumor vessel while a balloon catheter was inflated in the ICA to prevent inadvertent embolization of normal vessels and travel of embolic material into the intracranial circulation [19].

Initial approaches for embolization of lesions extending past the lateral wall of the nasopharynx often involved the delivery of small embolic particles, such as PVA ranging from 150 to 250 mm or Gelfoam [1,20]. Inadvertent nonselective embolization can occur with such small particles and requires simultaneous transient occlusion of the ICA, protecting the intracranial vasculature while allowing for adequate penetration into the tumor bed [20]. Because of difficulty in accessing distal vessels of the tumor bed, small particles allow for deeper penetration and embolization of distal vessels but are accompanied by a higher risk profile of intracranial emboli and stroke through extracranial-to-intracranial anastomoses [11]. To limit the potential complications associated with embolizations with any agent, it is recommended to always inject a small amount of contrast material intratumorally just before delivering the embolizing agent to minimize the risk of inadvertent vessel embolization and stroke [21].

Currently, direct tumoral puncture can be a safe and efficient method of embolization of the nidus of a JNA. Although direct tumor puncture can allow for better penetration of lower grade JNA lesions, lesions with infratemporal, pterygomaxillary, and intracranial extension may not be adequately visualized to allow for direct tumor puncture [22]. Although the use of embolic agents including NBCA, Gelfoam, and PVA are effective in direct puncture, complications such as stroke still can occur due to difficulty visualizing vessel anastomoses more proximal to the target vasculature [5,17,23].

The direct tumoral puncture has the benefit of deeper tumor bed penetration and embolization but may increase the risk profile for more complex lesions. For such complex lesions, there has also been a description of trimodal embolization for a grade IV lesion for which percutaneous, direct tumoral puncture and transarterial routes were used [24]. The use of multimodal embolization techniques through multiple catheters in a staged fashion can achieve adequate preoperative embolization and 15% more tumor devascularization compared to utilizing a single approach [24]. This is similar to the outcomes seen with our embolization of grades IIIa and IVa lesions. Having the ability to safely embolize >80% of the tumor preoperatively allowed for a safer resection plane.

Embolic agents

With respect to the choice of embolic agents, we found that Onyx was effective and safe in our series of JNA embolization procedures. Several of the aforementioned reports demonstrate effectiveness with the use of agents such as NBCA and PVA [7-9]. However, those agents have several limitations and risks. NBCA is a fast-acting, permanent, nonabsorbable liquid adhesive that needs to be administered in a quick and controlled fashion and also limits its penetrating capabilities into deeper tumor vasculature [25]. NBCA also carries the risk of inadvertent feeding vessel and parent artery avulsion during attempts to remove the microcatheter after successful embolization due to its tendency to adhere to the microcatheter [25]. PVA is inert and nonabsorbable but has surface irregularities and particle size variations. It also swells, contributing to further variation in individual particle size when inundated in contrast agent. Thus, PVA may aggregate and clump together, carrying the possibility of catheter and parent and proximal vessel occlusions and risk of complications as well as a failure of treatment [26]. Onyx, like NBCA, is capable of deep penetration into the tumor bed but, due to the lack of a fast polymerizing nature like NBCA, allows for safer catheter withdrawal and less risk of feeding vessel avulsion [27]. Onyx has relatively slower rates of precipitation and polymerization, which allows for more controlled injections and tumor bed penetration [28]. Furthermore, Gao et al. found that intraoperative blood loss and rates of intraoperative blood transfusions were much lower when Onyx was utilized as the embolic material over other particulate agents [23].

The extent of devascularization of JNAs

The mean percentage of devascularization of our five patients was 86%. Elhammady et al. utilized the same ImageJ software to determine the percentage of devascularization describes a 77%-93% mean obliteration rate after endovascular treatment of 10 patients with Onyx, which is comparable to our results [16]. Rosenbaum-Halevi et al. also describe a case series of nine patients that underwent pre-resection JNA embolizations with Onyx with a mean tumor volume embolization/devascularization of 84%, which is also similar to our results [19]. Thus, Onyx is shown to be an effective agent yielding high percentages of devascularization of tumors without a high risk of complications or neurological injury.

Limitations

This study has some limitations. The blood losses reported in the study are estimative and approximations obtained from intraoperative notes and anesthesia records. Furthermore, there are only five patients included in this series, which does not allow for sufficient statistical power and does not allow for generalization about whether Onyx embolization decreases intraoperative blood loss and is significantly effective. However, it was noticed in the surgical operative notes of all JNA tumors resected that embolized sections of the tumor allowed for an easier dissection plane around the border of the tumor and safe debulking of grades IIIa and IVa lesions. Finally, although in our series of JNA, transarterial Onyx embolization was found to be effective, some tumors are angiographically occult and may not be effectively embolized for surgical resection [29].

## Conclusions

In our experience, transarterial embolization of Fisch grades II-IVa JNAs was feasible and safely accomplished. Onyx embolic material can be delivered safely to gain adequate tumor penetration through transarterial routes for JNAs that extend beyond the sphenopalatine region. Although either transarterial methods can be applied to achieve the goal of hemostasis for Fisch grades II-IIIa lesions, higher-grade lesions may warrant transarterial embolization with embolic materials that allow for tumor bed penetration, such as Onyx.
